# Cut From the Same Cloth: Bipolar disorder and Frontotemporal Dementia – Apropos a Clinical Case

**DOI:** 10.1192/j.eurpsy.2023.1484

**Published:** 2023-07-19

**Authors:** S. Jesus, A. R. Costa, M. Almeida, P. Garrido

**Affiliations:** Departamento de Psiquiatria e Saúde Mental, Centro Hospitalar do Baixo Vouga, Aveiro, Portugal

## Abstract

**Introduction:**

Mood disorders have been reported in the literature as a risk factor for developing cognitive deficits. Bipolar disorder (BD) and Frontotemporal Dementia (FTD) share many common features, often presenting as a differential diagnostic challenge to the clinician. The clinical features of mania, such as euphoria, hyper-sexuality and difficulties in impulse control can mimic the impaired judgment and loss of inhibition seen in FTD. Depressive features such as anhedonia and social isolation can mimic apathy associated with FTD. Of the various subtypes, the behavioural-variant of FTD (bvFTD) is most similar to a manic episode.

**Objectives:**

The authors aim to explore the relationship between BD and FTD, and the implications in differential diagnosis, treatment and prognosis with recourse to a clinical case example.

**Methods:**

A non-systematized review of pertinent literature on the topic with focus on that which is most relevant to the theme was included. The authors present a clinical case of 55 year-old female with history of BD who was hospitalized in the context of a depressive episode with suicidal ideation and disorganized behaviour.

**Results:**

It is not uncommon for patients with bvFTD to be initially diagnosed with BD, whereas on the other hand, patients presenting in late with an inaugural manic episode are considered to have dementia. The literature also reports that patients with BD appear to be at increased risk of a later FTD diagnosis, further contributing the diagnostic difficulties. Core symptoms that present in mood disorders, also make-up the clinical picture of FTD, and vice versa. Correct diagnosis is imperative as early-intervention may have significant impact on prognosis of the clinical pictures. The patient underwent complementary diagnostic imaging testing with magnetic resonance imaging, which documented atrophy in the fronto-temporal regions which were not detected on previous exams, thus strongly suggesting a FTD diagnosis in a patient with history of BD.

**Conclusions:**

The literature establishes, especially through various case reports, an apparent clinical overlap between FTD and mood disorders. A multifaceted connection between BD and FTD appears to exist, with clinical and genetic similarities having been described, although further studies are merited demonstrating this relationship. The clinical case highlights the challenges in FTD diagnosis in a patient with prior history of a mood disorder, especially BD, as well as demonstrating the difficult task in establishing a differential diagnosis between the two conditions when the mood disorder presents late in life. The clinician is alerted to the mimicry between the two conditions, taking into account the possibility of a FTD diagnosis in patients with history of BD presenting with unexpected cognitive and behavioural decline.

**Disclosure of Interest:**

None Declared

